# Multiple Roles in Neuroprotection for the Exercise Derived Myokine Irisin

**DOI:** 10.3389/fnagi.2021.649929

**Published:** 2021-04-16

**Authors:** Mohammad Jodeiri Farshbaf, Karina Alviña

**Affiliations:** ^1^Department of Biological Sciences, Texas Tech University, Lubbock, TX, United States; ^2^Department of Neuroscience, University of Florida, Gainesville, FL, United States

**Keywords:** neuroprotection, myokine, exercise, skeletal muscle, brain

## Abstract

Exercise has multiple beneficial effects on health including decreasing the risk of neurodegenerative diseases. Such effects are thought to be mediated (at least in part) by myokines, a collection of cytokines and other small proteins released from skeletal muscles. As an endocrine organ, skeletal muscle synthesizes and secretes a wide range of myokines which contribute to different functions in different organs, including the brain. One such myokine is the recently discovered protein Irisin, which is secreted into circulation from skeletal muscle during exercise from its membrane bound precursor Fibronectin type III domain-containing protein 5 (FNDC5). Irisin contributes to metabolic processes such as glucose homeostasis and browning of white adipose tissue. Irisin also crosses the blood brain barrier and initiates a neuroprotective genetic program in the hippocampus that culminates with increased expression of brain derived neurotrophic factor (BDNF). Furthermore, exercise and FNDC5/Irisin have been shown to have several neuroprotective effects against injuries in ischemia and neurodegenerative disease models, including Alzheimer’s disease. In addition, Irisin has anxiolytic and antidepressant effects. In this review we present and summarize recent findings on the multiple effects of Irisin on neural function, including signaling pathways and mechanisms involved. We also discuss how exercise can positively influence brain function and mental health via the “skeletal muscle-brain axis.” While there are still many unanswered questions, we put forward the idea that Irisin is a potentially essential mediator of the skeletal muscle-brain crosstalk.

## Introduction

Over millions of years humans have evolved and developed the ability to run on two legs. Massive skeletal muscle development was paralleled with crucial changes in the brain (Noakes and Spedding, 2012), to facilitate the complex cognitive processing needed for running (Mendoza and Merchant, 2014). Thus, running did not only help skeletal muscle development but also promoted maturation of the brain (Schulkin, 2016). Evolutionary findings indicate a powerful crosstalk between muscle and brain. Several studies have provided evidence that exercise has beneficial effects on cognition and mental health in humans and also rodents models (Pietropaolo et al., 2008; Fuss et al., 2010; Mattson, 2012; Deslandes, 2014), including reducing the risk of developing neurodegenerative disorders associated with aging (Ahlskog et al., 2011). Exercise also improves several basic physiological functions such as appetite and sleep (Kline, 2014; Thackray et al., 2016).

Skeletal muscle tissue adapts to external stimuli, it has a high energy demand and actively controls metabolic homeostasis (Carbone et al., 2012). Skeletal muscle cells can also function as secretory organs in response to different stimuli such as exercise and cold exposure (Cannon and Kluger, 1983; Sepa-Kishi et al., 2017). Myokines are a group of cytokines and other small proteins that are synthesized and secreted by skeletal muscle cells upon muscular contraction. Myokines participate in building communications channels between skeletal muscle and other tissues (see reviews Trayhurn et al., 2011; Pedersen and Hojman, 2012). While the expression and secretion of myokines are mostly induced by muscle contraction, baseline expression of myokines in skeletal muscle conducts differentiation, proliferation, and regeneration of muscle cells (Clow and Jasmin, 2010; Broholm et al., 2011; Petersson et al., 2013).

Fibronectin type III domain-containing protein 5 (FNDC5) is a transmembrane protein that was discovered in 2002 (Ferrer-Martinez et al., 2002; Teufel et al., 2002). Initially, the main functions of FNDC5 were determined to be myocyte differentiation and development (Ferrer-Martinez et al., 2002). Then in 2012, it was discovered that FNDC5 is cleaved by enzymatic action, and a segment of the protein becomes a secreted peptide named Irisin (Bostrom et al., 2012). Interestingly, Irisin secretion is potently induced by exercise (Bostrom et al., 2012; Wrann et al., 2013). Furthermore, recent findings have shown that Irisin influences expression of different neuronal genes that mediate neuronal plasticity (Wrann et al., 2013; Forouzanfar et al., 2015), and that can potentially counteract neurodegeneration (Lourenco et al., 2019).

In this review we summarize recent finding on different signaling pathways and neural processes that are affected by the myokine FNDC5/Irisin in the brain. We specifically focus on Irisin as an important mediator of the communication between skeletal muscle and the brain. We also discuss the clinical significance of exercise and its impact on neurological disorders and mental health.

## Impact of Exercise on Skeletal Muscle

The Skeletal muscle has been shown to change in structure/function in response to exercise (Hoppeler, 1986; Matsakas et al., 2012). As a heterogenous tissue, skeletal muscle is composed of different fibers. Based on metabolic activity, muscle fiber types include oxidative (slow twitch, type I), oxidative-glycolytic (fast twitch, type IIa) and glycolytic (fast twitch, type IIX/IIb) in rodents and humans (Bottinelli et al., 1994; Schiaffino and Reggiani, 1994). Type I fibers have abundant mitochondria, exhibit high aerobic metabolism and oxidative enzymes activity. Type IIX/IIb fibers depend on glycolysis and anaerobic metabolism (Pette, 1985). As an intermediate, type IIa fibers use both aerobic and anaerobic metabolism for generating energy (Bourdeau Julien et al., 2018). The type of activity and exercise induce transition of fibers from fast-to-slow or slow-to-fast (Pette and Staron, 2000).

Additionally, skeletal muscles can act as secretory organs (Pedersen and Hojman, 2012; Egan and Zierath, 2013). As such, the skeletal muscle synthesizes and secretes cytokines and other peptides collectively named “myokines” (Pedersen and Hojman, 2012). Myokines employ autocrine, paracrine, and/or endocrine strategies to mediate different functions at cellular level on skeletal muscles and other organs (Pedersen and Hojman, 2012; Huh, 2018). For example, myokines control differentiation, growth, and regeneration of muscle fibers through autocrine mechanism (McPherron et al., 1997; Burks and Cohn, 2011; Petersson et al., 2013). Further, evidence shows that endocrine mechanisms of action mediate the functional association between skeletal muscle and other organs, including the brain. From adaptation to physiological changes to protecting cellular function depend on myokines acting in an endocrine fashion (Mohr et al., 1997; Marasco et al., 2018). Different factors such as exercise, pathological conditions and hormonal level stimulate myokine synthesis and secretion (Steensberg et al., 2000; Ciaraldi et al., 2016; Roberts et al., 2017). Exercise (aerobic and resistance) stimulates synthesis and secretion of different myokines into the circulatory system (Table 1).

**TABLE 1:** Myokines and type of exercise that induce their secretion in rodents and humans.

Myokine	Type of exercise	Species	References
Irisin	Aerobic/Resistance	Rodents/Human	Wrann et al., 2013; Huh et al., 2015; Tsuchiya et al., 2015; Tine Kartinah et al., 2018
Fibroblast growth factor-21 (FGF21)	Aerobic/Resistance	Rodents/Human	Kim et al., 2013; Kim and Song, 2017
Vascular endothelial growth factor (VEGF)	Aerobic/Resistance	Rodents/Human	Lloyd et al., 2003; Gavin et al., 2007; Delavar et al., 2014
Growth differentiation factor 15 (GDF-15)	Aerobic	Human	Kleinert et al., 2018
Brain derived neurotrophic factor (BDNF)	Aerobic/Resistance	Rodents/Human	Jimenez-Maldonado et al., 2014; Church et al., 2016; Kim et al., 2020; Nilsson et al., 2020
Decorin	Aerobic/Resistance	Rodents/Human	Kanzleiter et al., 2014; Marqueti et al., 2018
Leukemia inhibitory factor (LIF)	Aerobic/Resistance	Rodents/Human	Broholm and Pedersen, 2010
Interleukin-6 (IL-6)	Aerobic/Resistance	Rodents/Human	Trenerry et al., 2011; Chowdhury et al., 2020
Interleukin-15 (IL-15)	Aerobic/Resistance	Rodents/Human	Quinn and Anderson, 2011
Meteorin-like (Metrnl)	Aerobic	Rodents/Human	Rao et al., 2014; Bae, 2018
Myonectin	Aerobic	Rodents/Human	Otaka et al., 2018; Pourranjbar et al., 2018

The Central nervous system (CNS) is strongly influenced by myokines (Qin et al., 2013; Briken et al., 2016). For example, skeletal muscle derived BDNF, IL-6, FGF-21 and Irisin can cross the blood-brain barrier (BBB) and directly affect the neural function and activity, influencing synaptic plasticity and protecting neurons against degeneration (Banks et al., 1994; Pan et al., 1998; Hsuchou et al., 2007; Ruan et al., 2019). Furthermore, Irisin is a recently discovered exercise-induced myokine that controls a wide range of cellular signaling in different organs. Irisin is cleaved from the transmembrane protein FNDC5 during aerobic and/or resistance exercise in rodents and humans (Kraemer et al., 2014; Kim et al., 2015; Zhao et al., 2017; Shirvani and Rahmati-Ahmadabad, 2019). In mice, 72% of Irisin is derived from skeletal muscle and 28% from adipose tissue (Bostrom et al., 2012). In the next section we will focus on muscle derived FNDC5/Irisin and how exercise regulates its expression. We will also review signaling pathways related to FNDC5/Irisin in skeletal muscle and CNS.

## The Myokine Irisin

### Structure

**FIGURE 1:**
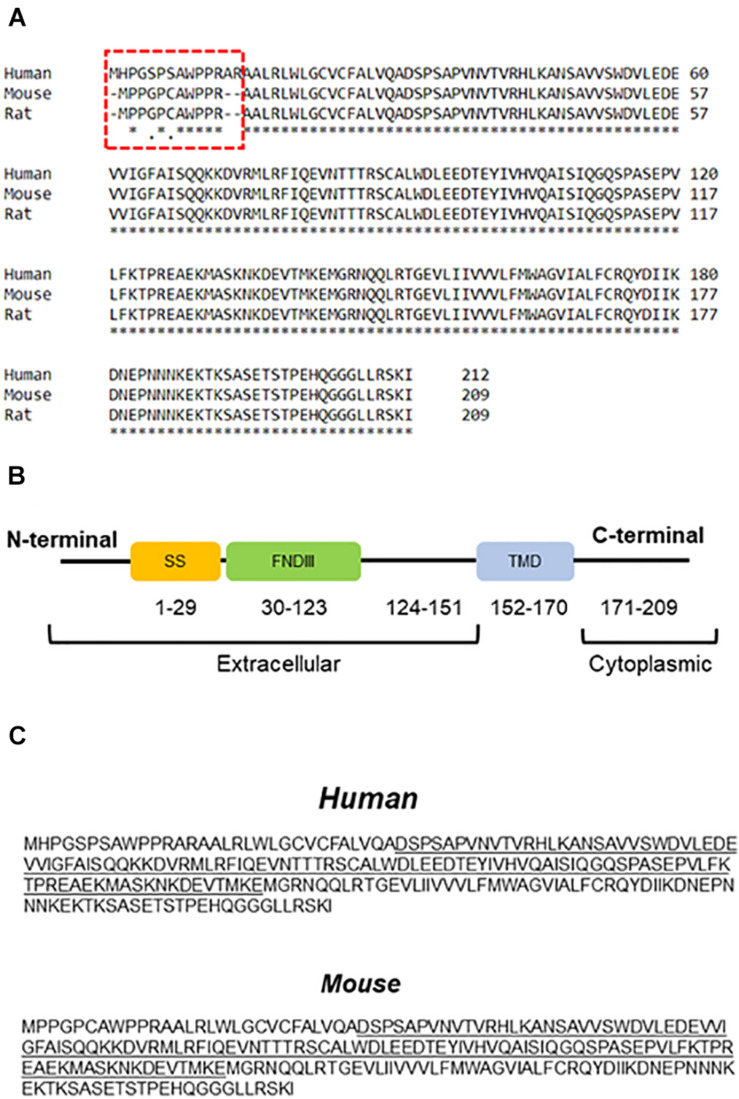
Amino acid sequence and protein structure of FNDC5. **(A)** Comparison between FNDC5 amino acid sequences from different species. The highlighted region depicts N-terminus region of the protein. **(B)** Schematic structure of FNDC5. SS, signal sequence; FNDIII, fibronectin domain III; TMD, transmembrane domain. **(C)** FNDC5 and Irisin (underlined) amino acid sequence in human and mouse.

In 2002, two research groups introduced FNDC5 as a protein involved in myoblast differentiation, initially named Peroxisomal protein (PeP) (Ferrer-Martinez et al., 2002; Teufel et al., 2002). Early analyses indicated high expression of FNDC5 in skeletal muscle, heart, and brain (Ferrer-Martinez et al., 2002). FNDC5 is a type I membrane protein that has 209 and 212 amino acids in rodents and humans, respectively, with the main difference located in the extracellular N-terminal segment (Figure 1A). FNDC5 is composed of an N-terminal signal sequence, a fibronectin III (FNIII) domain, an unknown domain, a hydrophobic transmembrane domain, and a cytoplasmic C-terminal (Bostrom et al., 2012; Schumacher et al., 2013) (Figure 1B). The N-terminal signal peptide is an endoplasmic reticulum (ER) transport signal needed for FNDC5 maturation and cleavage (Nie and Liu, 2017).

Different stimuli such as exercise and cold exposure induce FNDC5 cleavage at the ectodomain portion (Bostrom et al., 2012; Lee et al., 2014). The cleaved part produces a soluble segment named Irisin that consists of 112 amino acids. Protein sequence analysis of FNDC5 shows the cleavage site at position 28–29 in rodents (Figure 1C). However, in humans, the cleavage site is predicted in positions 31–32 (Figure 1C). Further proteolytic cleavage is performed between positions 140–141 (Nie and Liu, 2017). Therefore, the secreted part Irisin includes the N-terminal, FNIII domain and C-terminal tail (residues 29–140). Irisin molecular weight is ∼12 kD however analysis by X-ray crystallography showed dimerization of Irisin through FNIII domain (Schumacher et al., 2013). Other studies have shown a range of molecular weight from ∼12 to ∼35 kD (Bostrom et al., 2012; Nie and Liu, 2017). This difference may represent dimerization and post-translational modifications such as *N*-glycosylation, which can alter the number and/or structure of glycans attached to the protein (Nie and Liu, 2017; Korta et al., 2019). For example, after complete deglycosylation, Irisin was detected at ∼12 kD in human plasma (Jedrychowski et al., 2015) or 15 kDa in mice (Zhang et al., 2014). Other studies however, showed that deglycosylation reduced Irisin molecular weight below 12 kD (Schumacher et al., 2013; Nie and Liu, 2017). The signal sequence has a crucial role in post-translation modification of FNDC5. Removing the signal peptide, N-terminal, or cleavage site has significant effects on FNDC5 glycosylation, process that influences the stability of the protein and secretion Irisin (Nie and Liu, 2017). *N*-glycosylation of Irisin may have an important role in browning of white adipose tissue (Zhang et al., 2014). Therefore, while many important details are still unclear, glycosylation of Irisin not only changes its molecular mass but also can influence its stability and activity.

Several differences between rodent and human FNDC5 were found using genomic and transcriptomic analyses. For example, human *fndc5* (gene) has an ATA as a start codon instead of ATG as in rodents (Raschke et al., 2013). Further, the human *fndc5* transcript (with ATA as a start codon) has been reported to translated into protein with low efficiency (Raschke et al., 2013). In genes with non-AUG start codon, hairpin loop formation in downstream of the start codon increases the efficiency of translation (Kozak, 1990). Recently, it was shown that there are several transcripts for human *fndc5* in skeletal muscle, due to non-canonical start codon (Albrecht et al., 2020). Further, the first ATG codon is downstream of the ATA codon and could be used as start codon to translate into the short version of FNDC5 (Raschke et al., 2013).

**FIGURE 2:**
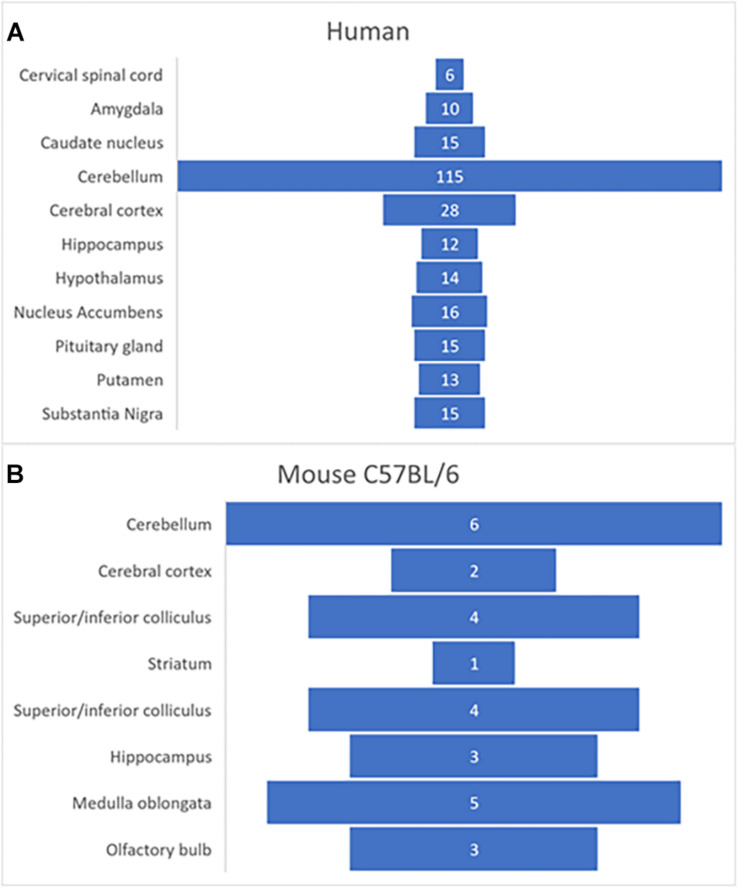
Expression of *fndc5* gene in human **(A)** and C57BL/6 mouse **(B)** brain. Data was obtained from publicly available databases from GTEx Consortium (2015; Perez-Lopez et al., 2018). The numbers represent TPM (transcripts per million) units.

Using transcriptome profiling through RNA-sequencing (RNA-Seq), FNDC5 expression was analyzed in different tissues from both male and female juvenile mice (C57BL/6 strain), and humans (Functional Annotation of the Mammalian Genome/Genotype-Tissue Expression Project) (GTEx Consortium, 2015)^1^. Transcriptome analysis showed that in humans and mice the gene *fndc5/Fndc5* has its highest expression in skeletal muscle, heart, and several regions in the brain, including hippocampus, cortex, medulla oblongata and in particularly high level, the human cerebellum (Figure 2). This pattern raises interesting questions about the possible role of FNDC5/Irisin in modulating the essential cerebellar function of motor control and planning, though future research is needed to expand these findings. Furthermore, this differential expression of FNDC5/Irisin might be regulated through various signaling pathways in different tissues and possibly in an activity dependent manner. The following section discussed recent advances on the mechanisms involved in regulating FNDC5/Irisin expression.

### Regulation of the Expression of FNDC5/Irisin

#### Expression in Skeletal Muscle

Both the expression of FNDC5 protein and secretion of Irisin are regulated by different external and internal stimuli, although basal expression of *Fndc5* (gene) is different in different types of muscle fibers. For example, FNDC5 has a higher expression in slow twitch fibers in comparison to fast type fibers, and after 3 weeks of aerobic exercise (i.e., running wheel), FNDC5/Irisin expression increased in both fiber types (Roca-Rivada et al., 2013). Further studies have shown that FNDC5 expression is indeed modulated by the type and duration of exercise (Bostrom et al., 2012; Ellefsen et al., 2014; Tiano et al., 2015).

While the skeletal muscle showed abundant expression of FNDC5 in both rodents and humans (Bostrom et al., 2012; Huh et al., 2012), other studies have shown that different tissues from the CNS to placenta express FNDC5 (Varela-Rodriguez et al., 2016). However, the secretion of Irisin is restricted to few tissues. Namely, the majority of Irisin is secreted into the peripheral blood from skeletal muscle during exercise (Bostrom et al., 2012), while another important source are the subcutaneous and visceral adipose tissues (Bostrom et al., 2012; Roca-Rivada et al., 2013). Therefore, Irisin is not only secreted as a myokine from skeletal muscles, but also it has the potential to be released into the peripheral blood as an adipokine.

Peroxisome proliferator-activated receptor gamma coactivator 1-alpha (PGC-1α) is the main regulator of FNDC5 in skeletal muscles in rodents and humans (Bostrom et al., 2012; Huh et al., 2014b). As co-activator, PGC-1α interacts with a wide range of transcription factors and it is expressed in high energy demand tissues such as skeletal muscle, heart, and brain (Lin et al., 2002; Buroker et al., 2008; Katsouri et al., 2016). PGC-1α is involved in different responses to distinct stimuli, controls mitochondrial biogenesis and glucose/fatty acid metabolism (Lehman et al., 2000; Finck and Kelly, 2006). In response to exercise, PGC-1α expression is increased in skeletal muscle which expedites mitochondrial biogenesis and switching of fast to slow twitch fibers (Lin et al., 2002; Koves et al., 2005).

Exercise induces the expression of PGC-1α in skeletal muscles, in rodents and humans (Little et al., 2010; Wrann et al., 2013). During exercise, muscle contraction needs Ca^2+^ influx to function properly. Rising Ca^2+^ influx in skeletal muscle augments PGC-1α expression and activity (Baar et al., 2002). Therefore, the expression level of PGC-1α could be a marker for exercise and skeletal muscle contraction (Egan et al., 2010; Egan and Zierath, 2013; Brandt et al., 2017). Furthermore, increasing PGC-1α in skeletal muscle in response to exercise might be a strategy for balancing energy influx (Olesen et al., 2010).

PGC-1α binds and/or activates transcription factors that differentially induce FNDC5 expression (for review see Handschin and Spiegelman, 2006). For example, endurance exercise (voluntary running wheel), induces FNDC5 expression in skeletal muscles (quadriceps) through PGC-1α/estrogen-related receptor alpha (ERRα) pathway (Wrann et al., 2013). Further, it has been shown that cAMP response element-binding protein (CREB) act as a transcription factor that controls FNDC5 expression in C2C12 myotubes by binding to PGC-1α (Yang et al., 2018). Importantly, aerobic exercise can activate CREB in skeletal muscle (Popov et al., 2019). Activation of cAMP signaling during exercise in skeletal muscle activates CREB in response to metabolic adaptation (Berdeaux and Hutchins, 2019).

Treatment with retinoic acid (RA) increases FNDC5 expression in differentiated C2C12 myocytes (Amengual et al., 2018). RA is a natural ligand for retinoid X receptor (RXR). RXR is a ligand−activated transcription factor and binds to retinoic acid−responsive elements (RARE) in the regulatory sequences of genes dependent and independent of PGC-1α (le Maire et al., 2012). In C2C12 myocytes, induction of FNDC5 by RA is independent from PGC-1α (Amengual et al., 2018). Mouse *Fndc5* gene has an RXR binding site in the promoter region (Seifi et al., 2014). Further investigations are needed to uncover the regulatory role of RXR in inducing FNDC5 expression in skeletal muscles.

Different proteins and conditions can suppress the expression of PGC-1α/FNDC5 in skeletal muscle. For example, PGC-1α expression in skeletal muscle is reduced in humans and diabetic models in rodents (Jove et al., 2004; Mensink et al., 2007). Reduced expression of PGC-1α in diabetic models correlates with low Irisin level in serum, while FNDC5 expression in skeletal muscle does not change (Kurdiova et al., 2014). Similarly, the protein Mothers against decapentaplegic homolog 3 (SMAD3) can suppress PGC-1α and FNDC5 expression in C2C12 mouse myoblasts, while in *Smad3*^–/–^ mice, aerobic exercise increased serum Irisin in comparison to wild-type mice (Tiano et al., 2015). Myostatin is another factor which mediates the expression of PGC-1α/FNDC5 in skeletal muscle (Ge et al., 2017). Neutralizing myostatin in skeletal muscle increases PGC-1α and FNDC5 expressions at mRNA level (Shan et al., 2013). Myostatin is a myokine that inhibits myoblast differentiation, thus showing opposite actions to FNDC5 (Rios et al., 2001). Fasting for 48 h reduced FNDC5 expression in skeletal muscle and reduced circulating Irisin in serum, while intraperitoneal (i.p.) injection of insulin for 14 days showed similar effects on FNDC5 and Irisin levels in skeletal muscle and plasma (Varela-Rodriguez et al., 2016).

Membrane receptors transduce the information from external environment to the nucleus. Some receptors are internalized into the cytoplasm through endocytosis mechanism after binding to their ligands. Lourenco et al. (2019) showed that FNDC5/Irisin bound to unknown receptors on the surface of hippocampal neurons and astrocytes in culture. This evidence suggests a putative mechanism of endocytosis initiated by FNDC5/Irisin binding to its membrane receptor in the CNS, however, this is still unclear and such receptor has not been characterized. Further, Irisin uptake into A549 cells (human lung carcinoma cell line) is blocked by nystatin, an inhibitor of endocytosis (Chen et al., 2017). At the cellular level, Irisin influences endocytosis and exocytosis mechanisms differently. For example, in isolated mouse pancreatic islet cells, Irisin increased insulin secretion (i.e., exocytosis) in response to glucose (Zhang et al., 2018). Moreover, recombinant Irisin (50 nM) induced the secretion of lactate from primary human adipocytes in culture and stimulated glucose uptake in skeletal muscle (Huh et al., 2014a). Similarly, subcutaneous injection of Irisin increased glucose uptake in the brain which suggests it increased the endocytosis of glucose transporters (Wang and Pan, 2016).

In summary, diverse physiological conditions and environmental stimuli can modulate PGC-1α/FNDC5 pathway in skeletal muscle cells.

#### Expression of FNDC5/Irisin in the Brain

FNDC5 is highly expressed in several regions of the brain of rodents and humans (Figure 2). Interestingly, in rodent brains Irisin was detected in especially high level in Purkinje cells of the cerebellum and vestibular nuclei of the medulla oblongata, together with other areas such as hippocampus and cortex (Dun et al., 2013). In primates Irisin is highly expressed in hypothalamic arcuate and ventromedial nuclei (Wahab et al., 2019). However, the roles of endogenous FNDC5/Irisin in the CNS remain to be fully characterized.

Similar to skeletal muscle, FNDC5 expression in the brain is modulated by different physiological conditions and environmental stimuli. For example, hippocampal FNDC5 is increased with aerobic and resistance exercise in rodents (Wrann et al., 2013; Nokia et al., 2016), while it is not known if exercise modulates FNDC5 expression in other brain regions. Interestingly, environmental enrichment (EE) increases FNDC5 expression in the prefrontal cortex (Yu K. W. et al., 2020). Studies have shown EE protected neurons against injuries, induced neurogenesis, and increased brain activity (Zhang et al., 2017; Wang C. J. et al., 2019). Pathological conditions also change FNDC5 expression in different regions of the brain. In Alzheimer’s disease (AD) for example, FNDC5 expression is decreased not only in the hippocampus but also in prefrontal cortex (Lourenco et al., 2019). In individuals suffering from post-stroke depression, Irisin levels are low in the peripheral blood (Tu et al., 2018). In addition, Irisin administration into the lateral ventricle or hippocampus suppresses depression, acute stress-induced anxiety, and memory impairment (Siteneski et al., 2018; Jodeiri Farshbaf et al., 2020). Chronic treatment with insulin increases FNDC5 expression in the hypothalamus which is responsible for feeding behavior and energy homeostasis. More details of FNDC5/Irisin involvement in disease are in section “Role of FNDC5/Irisin in Neurological and Neuropsychiatric Disorders” of this review.

Details of how the expression of FNDC5/Irisin in the brain is regulated are emerging. It has been shown that PGC-1α controls FNDC5 expression in the hippocampus and primary neurons in culture (Wrann et al., 2013). In the hippocampus, this depends on the cAMP/PKA signaling pathway (Lourenco et al., 2019). Further, in the P19 cell line as well as differentiated C2C12 myocytes, RA treatment increased FNDC5 expression (Ostadsharif et al., 2011). Similarly, Lactate released from skeletal muscle during exercise induces FNDC5 expression in the hippocampus (El Hayek et al., 2019). Further analysis has shown a putative ERRα binding element (ERRE) located upstream to the *fndc5* promoter (Wrann et al., 2013). In primary cortical neurons, PGC-1α activated ERRα and increased FNDC5 expression, which then negatively fed back onto PGC-1α/ERRα (Wrann et al., 2013). Additional analysis of upstream regulatory sequences of the *fndc5* promoter could uncover putative binding elements that could be differentially regulated by activators, repressors, and transcription factors. This could elucidate different stimuli that could influence *fndc5* expression in different tissues and conditions.

### Signaling Pathways Involved and Function

#### Skeletal Muscle

FNDC5 was initially introduced as a regulator of myoblast differentiation (Ferrer-Martinez et al., 2002). *Fndc5* knockdown in muscle stem cells decreased the expression of myogenic genes and myotube formation without any effects on muscle growth (Lee et al., 2019). Recently, it was shown that inhibiting *Fndc5* expression induces autophagy and causes skeletal muscle atrophy (Pan et al., 2019). Further, loss-of-function mutation in the *Fndc5* gene in skeletal muscles decreases maximal oxygen consumption during aerobic exercise (Xiong et al., 2019). In addition, the mutant *Fndc5* mice showed higher glucose level after aerobic exercise in skeletal muscles while the blood glucose level did not change during fasting (Xiong et al., 2019). *Fndc5* overexpression in hind-limb muscle increases glycogen content in muscle and Irisin level in peripheral blood (Farrash et al., 2020). Modulation of FNDC5 level in skeletal muscle correlates with secreted Irisin level in mice and C2C12 cells (Tiano et al., 2015; Chen et al., 2019; Rodriguez Lanzi et al., 2020). Human clinical studies have shown that single nucleotide polymorphism in *Fndc5* is related to insulin level and sensitivity (Staiger et al., 2013).

Circulating Irisin acts via autocrine mechanisms on skeletal muscle fibers. For instance, Irisin induced muscle hypertrophy through activation of protein kinase B (also known as Akt), mammalian target of rapamycin (mTOR) and extracellular signal-regulated kinases (ERK) (Reza et al., 2017). Irisin-dependent hypertrophy was controlled by promoting protein synthesis and preventing protein turnover in skeletal muscle fibers. Interestingly, resistance exercise induced muscle hypertrophy as well through activation of mTOR signaling in mice and humans (Ogasawara et al., 2016; Song et al., 2017). This effect of resistance exercise on muscle mass through mTOR signaling pathway might be mediated by Irisin but this needs further investigation. In a recent human study, Irisin seemed to be a marker for improved muscle strength (Planella-Farrugia et al., 2019).

Irisin has been shown to control metabolism and energy expenditure in muscle cells. For instance, treating C2C12 cells with recombinant Irisin (5 nM) increased metabolism in a time and dose dependent manner. In the short-term (1–4 h), Irisin increased glycolytic metabolism whereas in long-term (24 h), incubation with recombinant Irisin resulted in increased mitochondrial biogenesis and oxidative metabolism (Vaughan et al., 2014). Further, recombinant Irisin increased glucose and lipid uptake in primary human skeletal muscle cells. Specifically, Irisin increased glucose transporter-4 (GLUT4) and hexokinase (HK) expression which are responsible for glucose uptake and use (Vaughan et al., 2014). Glycogen phosphorylase (PYGM), rate-limiting enzyme of glycogenolysis, was decreased by Irisin (50 nM) treatment in human skeletal muscle (Huh et al., 2014b).

One of the main downstream targets for FNDC5 in skeletal muscle cells is BDNF (Wrann et al., 2013; Wrann, 2015). The main roles for BDNF are regulating muscle regeneration and adapting myotubes to different metabolic conditions (Omura et al., 2005). BDNF also increases mitochondrial content and oxidative phosphorylation in C2C12 myotubes (Yang et al., 2019). Based on this evidence, Irisin may be important for metabolic flexibility and adaptation in skeletal muscles.

Both aerobic and resistance exercise types induce metabolic adaptation in skeletal muscles. AMP-activated protein kinase (AMPK) is the main cellular sensor for energy depletion and has a crucial role in metabolic adaptation (Winder and Hardie, 1996). Using high amounts of ATP during contraction increases AMP/ATP ratio in muscle cells which triggers AMPK activation and induces glucose uptake through GLUT-4 (Wright et al., 2004). In differentiated L6 muscle cells, acute treatment with Irisin (20–200 ng) increases glucose uptake through activation of AMPK after 15 min and up to 180 min (Wright et al., 2004). Injecting recombinant Irisin (100 μg/kg i.p. for 28 days) increases FNDC5 expression in skeletal muscle (Colaianni et al., 2015). Interestingly, acute exercise does not change AMPK level and activity in skeletal muscle, in fact acute exercise modulates glucose uptake and metabolism independent from AMPK pathway in skeletal muscle fibers (Mu et al., 2001; McConell et al., 2005). Acute exercise increases plasma levels of Irisin (∼100 ng/ml) in men without any change in women (Loffler et al., 2015). Therefore, Irisin may have an important role in inducing AMPK signaling pathway in skeletal muscle. Further, chronic activation of AMPK triggers mitochondrial biogenesis in skeletal muscle cells (Winder et al., 2000). Indeed, AMPK activates PGC-1α through direct or indirect pathways (Canto et al., 2009). A recent study showed that PGC-1α protein level was not changed after acute exercise while mRNA level greatly increased in skeletal muscle (Safdar et al., 2018). Interestingly, PGC-1α expression of mRNA and protein increased 1–3 h of recovery after acute exercise in skeletal muscles (Peng et al., 2017; Safdar et al., 2018). Chronic exercise training for 28 days gradually increased PGC-1α expression in skeletal muscle (Park et al., 2020). This evidence suggests that AMPK is upstream of FNDC5 expression in skeletal muscle cells (Lally et al., 2015). Therefore, AMPK may induce glucose uptake and glycolytic metabolism in skeletal muscle to restore ATP generation, and Irisin could be a mediator of metabolic adaptation in skeletal muscle during exercise. Interestingly, treatment for 24 h with Irisin in C2C12 cells increased oxygen consumption and ATP level (Vaughan et al., 2014). Therefore, it is possible to speculate that exercise and released Irisin impact glycolytic and oxidative metabolism during metabolic adaptation to maintain ATP levels. However, the signaling cascade including AMPK/PGC-1α/FNDC5 needs to be better understood in the context of skeletal muscle physiology.

#### Brain

Several studies have shown that the levels of FNDC5/Irisin can alter specific gene expression in neurons. For instance, the gene *Fndc5* can influence the differentiation of mouse embryonic stem cells to neural cells (Hashemi et al., 2013; Forouzanfar et al., 2015). Further, *Fndc5* overexpression induces the expression of *Bdnf*, Neuronal PAS Domain Protein 4 (*Npas4), cFos, and Arc* in primary cortical neurons (Wrann et al., 2013). Intriguingly, intracerebroventricular (i.c.v.) administration of Irisin (1 ng) in male mice increases BDNF mRNA level in the hippocampus but it decreases it in the prefrontal cortex (Siteneski et al., 2018).

An important question that remains unanswered is whether Irisin can control the expression of FNDC5 in the brain. A recent *in vivo* study showed that i.c.v. administration of Irisin leads to a short time decrease in FNDC5 expression in the prefrontal cortex and hippocampus after 1 h. However, the expression of FNDC5 was enhanced in the hippocampus without change in the prefrontal cortex 6 h after Irisin administration (Siteneski et al., 2018). The mechanisms of action for this change are not fully understood.

Additionally, Irisin can influence the function and activity of glial cells in different conditions. In pathological conditions, glial cells initiate the neuroinflammation responses to injuries through expression of cytokines such as IL-6 and tumor necrosis factor-α (TNF-α). Intravenous (I.V) injection of recombinant Irisin decreased the number of active microglia and TNF-α expression in the middle cerebral artery occlusion (MCAO) model (Li et al., 2017). Further, recombinant Irisin decreased TNF-α induced apoptosis in SH-SY5Y cells (Huang et al., 2020). Similarly, Irisin counteracted several changes induced in a model of streptozotocin-induced diabetes in mice. For instance, glial fibrillary acidic protein (GFAP), a marker for active astrocyte, was decreased in the hippocampus of Irisin-treated diabetic mice, change that was paralleled with a reduction in IL-6 level (Wang K. et al., 2019). Importantly, Irisin immunostaining has been observed in both neurons and glial cells (Aydin et al., 2014), including expression on the surface of astrocytes (Lourenco et al., 2019). Physiological concentration of Irisin (50–100 nM) increases proliferation of mouse H19-7 hippocampal cell lines through activating STAT3 signaling pathway (Moon et al., 2013). Further, in the focal cerebral ischemic stroke model, Irisin reduces the number of active microglia which protect neurons against inflammation (Li et al., 2017). This is important considering that neuroinflammation is one of the most important factors that accelerates brain injury in case of stroke (see review Jayaraj et al., 2019). In cultured astrocytes, Irisin increases ATP level and GLUT-4 expression (Wang and Pan, 2016).

Irisin was able to protect PC12 neuronal cells against cell death induced by oxygen/glucose deprivation (Li et al., 2017). Similarly, in an oxygen/glucose deprivation *in vitro* model, Irisin treatment protected neurons against apoptosis through an inflammatory signaling pathway (Peng et al., 2017). Furthermore, FNDC5 can bind to the N-terminus region of the amyloid precursor protein (APP) (Noda et al., 2018). APP is cleaved by β/γ-secretases and produces amyloid beta (Aβ) deposits which are one of the hallmark pathological signs of AD (Chow et al., 2010). Co-transfection of FNDC5 and APP into human embryonic kidney 293 (HEK293) cells decreased Aβ production (Noda et al., 2018). Therefore, this evidence suggests that FNDC5/Irisin may contribute to reduce APP cleavage and consequent Aβ secretion. More details of the involvement of FNDC5/Irisin in mechanism of neurodegenerative disorders are discussed in Section “Role of FNDC5/Irisin in Neurological and Neuropsychiatric Disorders” of this review.

Extensive research has shown that exercise increases memory and cognition (reviewed in Pedersen and Saltin, 2015; Vecchio et al., 2018). A recent report has shown that Irisin injected directly into the dentate gyrus (DG) in the hippocampus, increases long term potentiation (LTP) in male rats (Mohammadi et al., 2019). Moreover, blocking FNDC5/Irisin decreased the maximal LTP induced at Schaffer-CA1 synapses in mouse hippocampal slices (Lourenco et al., 2019). Inducing LTP correlates with synaptic plasticity and memory formation in the hippocampus, therefore, exercise may have beneficial effects on memory by altering mechanisms of synaptic plasticity, at least in the hippocampus. While these data are promising, the role of FNDC5/Irisin in mediating the beneficial effects of exercise on memory needs to be further investigated.

Different types of exercise induce the expression of FNDC5/Irisin in the CNS. Bostrom et al. (2012) showed that exercise (30 days of voluntary running or swimming for 14 days) can induce FNDC5 expression in skeletal muscles, and Irisin secretion into the peripheral blood in mice and human subjects. Further, FNDC5 expression also increased in the hippocampus of running mice, which results in increased BDNF expression (Wrann et al., 2013). These results confirmed previous studies showing that exercise increases BDNF level in the hippocampus (Neeper et al., 1995; Vaynman et al., 2004; Sleiman et al., 2016). This is relevant because hippocampal BDNF is associated with memory formation and cognition, and exercise and BDNF have crucial roles in inducing hippocampal neurogenesis, process that is also involved in memory function (reviewed in Spalding et al., 2013; Choi et al., 2018; Gonzalez et al., 2019). BDNF expression in the hippocampus is influenced by different factors that could mediate the connection between exercise and neurogenesis (Horowitz et al., 2020). For example, the PGC-1α/FNDC5/BDNF axis is activated in the hippocampus by voluntary running (Wrann et al., 2013). Moderate aerobic exercise increases PGC-1α/FNDC5/BDNF axis through activating AMPK signaling pathway in the hippocampus (Azimi et al., 2018). Interestingly, it has been shown that muscle contraction through electrical stimulation under anesthesia induces FNDC5/BDNF expression in the hippocampus without any change in skeletal muscle (Azimi et al., 2018).

As Irisin, lactate is released from skeletal muscle during exercise and can cross the BBB potentially acting as a neuroprotector (Berthet et al., 2009; Newman et al., 2011). In addition, lactate could be used as energy source by neurons (Quistorff et al., 2008). Interestingly, i.p. injection of lactate induces FNDC5/BDNF expression in the hippocampus (El Hayek et al., 2019). Similarly, Irisin i.p. injection (0.5 μg/g of body weight), used to imitate exercise, increases BDNF expression in the brain (Natalicchio et al., 2020). Therefore, this evidence indicates that Irisin (acting on unknown receptors in the brain) triggers BDNF expression and release, which could lead to memory and learning improvement. Furthermore, beside improving learning and memory, BDNF is also important for the brain to adapt metabolic challenges. Treatment with BDNF (10–40 ng/ml) increases mitochondrial biogenesis to maintain ATP level in hippocampal neurons in culture, by increasing the expression of PGC-1α (Cheng et al., 2012). This was also shown after chronic aerobic exercise in mice (8 weeks with a running wheel), which increased mitochondrial biogenesis through increasing PGC-1α in different regions of the brain (Steiner et al., 2011). In addition, BDNF induces glucose transportation in cortical neurons (Burkhalter et al., 2003), and exercise-induced hippocampal neurogenesis depends on BDNF controlled neuronal bioenergetic (reviewed in Mattson, 2012). One of the possibly involved signaling pathways, which has crucial role in memory and cognition, is the phosphoinositide 3-kinase (PI3K)/protein kinase B (Akt) signaling pathway (Shu et al., 2013). Curiously, treadmill exercise activates PI3K/Akt signaling pathway in the hippocampus (Wang and Baek, 2018). A recent study showed that human recombinant Irisin activates Akt in the brain (Li et al., 2017). PI3K/Akt signaling pathway is also activated by BDNF in neurons (Chen et al., 2013). Activation of PI3K/Akt controls glucose homeostasis, mitochondrial biogenesis, and integrity in neurons (Pearson-Leary et al., 2018). In other tissues such as injured lung alveoli, myocardial cell, and chondrocytes Irisin controls mitochondrial integrity and function (Chen et al., 2017; Wang et al., 2018, 2020). Nevertheless, the direct impact of the FNDC5/Irisin on mitochondrial integrity and function in neurons and/or glial cells remains to be fully elucidated.

A recent interesting discovery about Irisin function is related to autophagy (reviewed extensively in Pesce et al., 2020). Autophagy is an intracellular process that occurs to eliminate misfolded proteins and injured organelles, and in general to maintain the cellular homeostasis (reviewed in Glick et al., 2010). Autophagy is also involved in the pathology of neurodegenerative disorders such as AD, amyotrophic lateral sclerosis (ALS), and familial Parkinson’s disease (PD) (Nixon, 2013). Recent studies have shown that exercise stimulates autophagy in a variety of tissues, including skeletal muscles (Salminen and Vihko, 1984; Grumati et al., 2011), and several myokines have been recognized as autophagy regulators (Pesce et al., 2020). Irisin has been associated to autophagy induction in several cell types including myocytes, cardiomyocytes (Li et al., 2018), and hepatocytes (Bi et al., 2019, 2020). Further, Irisin increased autophagy in hepatic ischemia-reperfusion (IR) model and mitigated liver injury (Bi et al., 2020). Meanwhile, the knock down of FNDC5 results in autophagy and fatty acid oxidation impairment in mice, and enhancement of lipogenesis via the AMPK/mTOR pathway (Liu et al., 2016). However, future studies are needed to elucidate the potential role of FNDC5/Irisin in controlling autophagy in the brain. The implications for neurodegenerative disorders are extensive.

## Sex Dimorphism in Exercise and FNDC5/Irisin

Sexual dimorphism has been shown in a variety of physiological responses, pathological conditions and even in the effect of therapeutic agents. In neuroscience research, the role of sex hormones on neuronal activity and functions has not been studied in extensive detail. In addition to brain, skeletal muscles and myokines have also shown sex-dependent effects/functions in rodents and humans. In this section we review potential roles for estrogen, as one of the most prevalent circulating sex hormones, in modulating FNDC5/Irisin expression and function in skeletal muscle and CNS.

### Sex Hormones and Skeletal Muscles

Steroid sex hormones include estrogens, androgens, and progestogens. Aside from their well-known fundamental roles in reproduction, they contribute to a variety of other physiological responses and exert their actions via autocrine, endocrine, and paracrine pathways.

Progesterone is initially made from cholesterol during the steroid sex-hormone biosynthesis pathway. Progesterone is then converted to testosterone and androstenedione which is ultimately converted into estrogens and estrone (reviewed in Schiffer et al., 2019). 17β-estradiol (E2) is the most abundant estrogen in females and its level in peripheral blood change during the human menstrual cycle and rodent estrous cycles (Nilsson et al., 2015). Most of the effects of E2 are mediated by estrogen receptor (ER) α and β, both members of the nuclear receptor family and located intracellularly (reviewed in Hewitt and Korach, 2018). A second family of ERs has been found at the cell membrane (mER). These mERs signal via modulation of intracellular signaling pathways (Soltysik and Czekaj, 2013). Additionally, based on their sequence similarity to ERα, the orphan nuclear receptors called estrogen-related receptors α and β (ERRα and ERRβ) were first identified. Then a third type was found (ERRγ) and together these three receptors form the ERR subfamily of the steroid nuclear receptor superfamily (Giguere et al., 1988; Zhang and Teng, 2007). Activation of ERα induces the expression of ERRα (Hu et al., 2008). The ERRs contain DNA-binding domains that target the receptor to a specific DNA sequence (TCAAGGTCA) called the estrogen-related response element (ERRE) (Vanacker et al., 1999). Interestingly, ERR deficiency in skeletal muscle impairs regeneration after injury, while treatment with E2 stimulate its regeneration and growth (Velders et al., 2012; LaBarge et al., 2014).

Studies have shown ERRα controls metabolic homeostasis through regulating mitochondrial biogenesis, oxidative phosphorylation, and Krebs cycle (Huss et al., 2004; Dufour et al., 2011). ERRα is activated by binding to PGC-1α as a co-activator (Schreiber et al., 2004). *Fndc5* itself has an ERRE at 6 kb upstream of its promoter (Wrann et al., 2013). Therefore, it is possible to infer that ERRα activation or induction could influence FNDC5/Irisin levels. Indeed, studies have shown that the serum Irisin concentration is positively correlated with E2 levels in humans (Huh et al., 2012). Similarly, Irisin level in serum is lower in amenorrheic women in comparison to eumenorrheic young women (Singhal et al., 2014). In amenorrheic women E2 level is low because of less endogenous estrogen production). Furthermore, E2 is decreased during aging which correlates with Irisin decline and muscle mass loss (Brown, 2008; Huh et al., 2012). E2 may thus activate ERα/ERRα axis which could increase FNDC5 expression in skeletal muscle and Irisin level in serum. However, ovariectomy (OVX), which is the surgical removal of one or both ovaries, results in increased FNDC5 expression in skeletal muscle and Irisin level in serum (18 weeks after surgery) without affecting PGC-1α expression (Zugel et al., 2016). While these findings are unexpected based on previous human studies showing E2 and Irisin correlation, this discrepancy may be related to OVX-induced obesity (Asarian and Geary, 2006). In fact, circulating level of Irisin is higher in obese women (Zugel et al., 2016). Further, Irisin could also be secreted from adipose tissue in addition to skeletal muscle (Roca-Rivada et al., 2013).

E2 levels can also affect the main regulators of FNDC5 expression in skeletal muscle, namely AMPK and PGC-1α. For example, treatment with E2 increases AMPK phosphorylation in skeletal muscle and C2C12 cells (D’Eon et al., 2008; Rogers et al., 2009). Also, PGC-1α expression in skeletal muscle is decreased 9 weeks after OVX but recovered with E2 treatment (Capllonch-Amer et al., 2014). In human studies AMPK and PGC-1α expression in skeletal muscle changes over time after menopause. Studying samples from post menopause subjects showed that E2 treatment induces AMPK/PGC-1α axis in skeletal muscle only in early post menopause cases (less than 6 years post-menopause) (Park et al., 2017). Also, E2 treatment increases PGC-1α expression in skeletal muscle in men (Maher et al., 2010).

Skeletal muscle BDNF production has been shown to respond to metabolic adaptation in a sex-dependent manner (Yang et al., 2019). Further, plasma levels of BDNF and E2 have been shown to be positively correlated (Pluchino et al., 2009). Interestingly, a few exercise studies in rodents and humans have shown sex and gender-dependent differences. For example, aerobic exercise for 6 months does not result in increased Irisin level in young women with lower E2 level or amenorrheic subjects, in comparison to young women with normal menstrual period (Singhal et al., 2014). Furthermore, OVX mice subjected to exercise showed decreased endurance without changes in skeletal muscle mass, while mimicking estrous cycle with E2 injections recovered their deficiency for endurance exercise (Nagai et al., 2016). Based on this evidence, we could speculate that Irisin secretion/function may crosstalk with estradiol and/or ERs actions in female individuals. Our own data has shown interesting results when probing the anti-stress properties of irisin in male/female mice. We recently showed that Irisin successfully prevented the acute stress induced memory impairment observed in male mice only, with no protective effects in female mice (Jodeiri Farshbaf et al., 2020).

### Sex Hormones and FNDC5/Irisin in the Brain

Strong evidence of sex dimorphism in the CNS has been shown at different levels, ranging from molecular, circuit-base and behavior. To date, morphological differences, cognitive functions and different signaling pathways involved, they have all been shown to be influenced by sex. As discussed earlier, there have been a few previous reports showing a possible sex-specific relation between FNDC5/Irisin and related signaling pathways, and the effect of exercise. For instance, Wrann et al. (2013) showed that ERRα can modulate PGC-1α and FNDC5 expression in the hippocampus of male mice. Thus, treating primary cortical neurons with an ERRα inhibitor (XCT-790), decreased *fndc5* expression (Wrann et al., 2013). Remarkably, other studies have shown that PGC-1α can have sex-dependent effects. For example, nigral dopaminergic neurons of PGC-1α null mice display robust ultrastructural alterations in intracellular organelles such as ER and mitochondria, changes that result in increased vulnerability to α-synuclein specifically in male mice (Ciron et al., 2015). Also, PGC-1α deficiency leads to earlier onset and death in male animal models of ALS and male human subjects (Eschbach et al., 2013). Further, activation of the AMPK pathway protects memory function only in females in a mouse model of AD, while increasing dysfunction in males (DiTacchio et al., 2015).

As mentioned, PGC-1α binds to ERRα which is influenced by E2. OVX females show a reduced expression of PGC-1α in the brain (Zawada et al., 2015). In addition, mitochondrial function and dynamic are also affected in OVX females, effect that is reversed with E2 treatment (Yao et al., 2012). Treatment with E2 also increases PGC-1α expression and ameliorates mitochondrial dysfunction in Leber’s hereditary optic neuropathy (Giordano et al., 2011). Our studies have also shown that Irisin differentially rescues short-term memory in male versus female mice (Jodeiri Farshbaf et al., 2020). Different studies have shown that BDNF expression decreases in the brain after OVX, and E2 treatment leads to its recovery (Singh et al., 1995; Fortress et al., 2014). Other reports have corroborated these findings by showing that the protein level of BDNF is not increased in OVX female rats subjected to voluntary running wheel exercise for ∼2 weeks (Rashidy-Pour et al., 2019). This is relevant considering that after menopause, the risk of neurological and psychological disorders is increased in women (Brinton, 2008). Diverse studies have shown that E2 can have neuroprotective properties (Bonnefont et al., 1998; Platania et al., 2005). Further, astrocytes express α and β estrogen receptors (Kuo et al., 2010) and play a critical role in E2-induced neuroprotection (Spence et al., 2011). Several studies have shown that E2 induced the expression of various genes critical for neuroprotection such as nerve growth factor (NGF), BDNF and glial cell line derived neurotrophic factor (GDNF) (Platania et al., 2005; Xu et al., 2013). In addition to genomic changes, non-genomic pathways such as ERK and Akt mediate neuroprotection in response to E2 treatment (Nicole et al., 2001; Dhandapani et al., 2005). Therefore, E2 can mediate neuroprotection not only through neuronal pathways but also by mediating genomic and non-genomic mechanisms of action in astrocytes. An important question remains unanswered, does Irisin require glial cells to exert its putative neuroprotective functions?

Abruption of peripheral estrogens level and E2-dependent signaling pathways in the brain may play central role in increasing susceptibility of post-menopause women to neurological diseases. Because FNDC5/Irisin is one of the factors influenced by E2 levels, this suggests that FNDC5/Irisin might have an important role in protecting neurons against aging related CNS issues.

## Role of FNDC5/Irisin in Neurological and Neuropsychiatric Disorders

**FIGURE 3:**
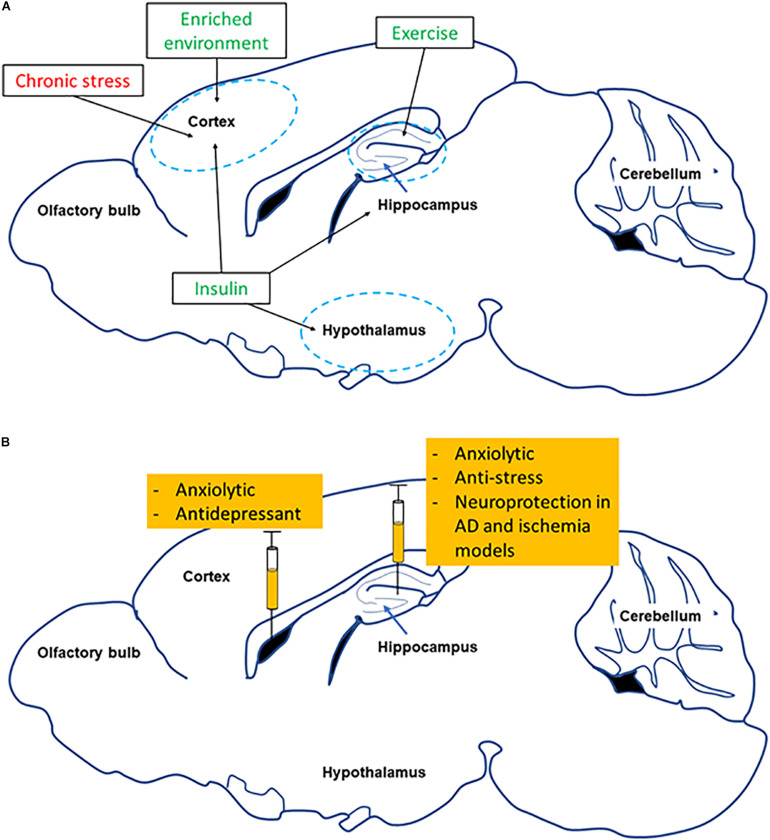
Summary of effects of changing FNDC5/Irisin levels in different brain regions. **(A)** Shows how several environmental factors lead to alterations in FNDC5/Irisin levels in different brain regions (red symbolizes reduction, green means increase). **(B)** Shows the effect of directly manipulating levels of FNDC5/Irisin on neurobehavior and neuroprotection.

Different studies have shown that exercise can have neuroprotective effects in conditions of neurodegenerative diseases (reviewed in Ahlskog et al., 2011). Further, in mental health disorders exercise can also be used as a therapeutic strategy for prevention and treatment (Zschucke et al., 2013). While exercise can change and modify the expression of several genes in different tissues that can eventually affect the CNS, we will focus primarily on the skeletal muscle-brain axis and the potential for FNDC5/Irisin as a mediator of beneficial effects (Figure 3 and following sections).

### FNDC5/Irisin in Neurological Diseases

Exercise induced neuroprotection has been demonstrated in rodents and humans. However, the underlying mechanisms and pathways involved are far from fully understood. In a landmark study, Lourenco et al., showed that the expression of FNDC5 in the hippocampus is decreased in both AD patients and AD mouse models (Lourenco et al., 2019). AD is the most prevalent neurodegenerative disorder in elderly people (Prince et al., 2013), therefore there are tremendous efforts dedicated to device therapeutic alternatives to delay or counteract AD’s devastating consequences. It has been also shown that AD patients have less circulating Irisin in their cerebrospinal fluid (CSF) (Lourenco et al., 2019, 2020). FNDC5 also decreases Aβ production by binding to the N-terminus of APP (Noda et al., 2018). While Aβ oligomers decrease the expression of components of the PGC-1α/FNDC5/BDNF axis in neuro-2a (n2a) cells, mice, and humans (Xia et al., 2017; Lourenco et al., 2019).

Several studies have shown that exercise can delay or improve the memory decline in AD patients (Winchester et al., 2013; Jia et al., 2019). Lourenco et al. (2019) showed that using neutralizing circulating Irisin antibodies suppressed exercise-induced memory improvement in AD mouse model. Furthermore, in PD exercise not only improved memory deficit but also has impacted psychological indicators such as depression, anxiety, and psychosis (Tanaka et al., 2009; Petzinger et al., 2013). While no studies have directly indicated a role for FNDC5/Irisin in alleviating PD pathology, it has been shown that PGC-1α and BDNF (upstream and downstream from FNDC5/Irisin, respectively) can alleviate PD symptoms (St-Pierre et al., 2006; Palasz et al., 2020). For example, aerobic exercise increases BNDF protein level in the substantia nigra of a PD model mouse, which leads to protection against further degeneration (Lau et al., 2011). BDNF level in peripheral blood and brain is decreased in PD rodent models and humans (Wang et al., 2016; Lin et al., 2017). In addition, PGC-1α expression is reduced in human PD brain samples (Zheng et al., 2010). Further, in animal models of PD overexpressing PGC-1α levels can result in suppression, while knocking out PGC-1α can accelerate neurodegeneration (St-Pierre et al., 2006; Jiang et al., 2016). Based on this evidence, FNDC5 could have an important role in alleviating PD pathology.

Exercise is showing promise as an effective strategy to protect neurons against degeneration not only in AD and PD, but also in Huntington’s disease (HD). Expansion of CAG repeats in Huntingtin (*HTT*) gene leads to neurodegeneration in striatum in HD (Vonsattel and DiFiglia, 1998). As in AD and PD, BDNF level is decreased in HD mouse model and human samples (Zuccato et al., 2001, 2005). Voluntary running wheel increases BDNF level in striatum and frontal cortex of HD mice, which results in improvement of behavioral deficits and cognitive decline (Pang et al., 2006). In the R6/2 HD model, PGC-1α expression is suppressed which leads to increased neurodegeneration in the striatum (Cui et al., 2006). Overexpression of PGC-1α in striatum and cortex ameliorate HD pathology in mice (Tsunemi et al., 2012). Interestingly, in HD model PGC-1α expression is decreased in skeletal muscle (Chaturvedi et al., 2009). FNDC5 expression and Irisin secretion from skeletal muscle are processes downstream from PGC-1α expression, it is currently unknown how this is regulated in skeletal muscle of PD subjects. Taken together, these results suggest that FNDC5/Irisin could have a potential role in rescuing HD pathology and symptoms.

FNDC5/Irisin has also shown neuroprotective properties in conditions of ischemia. In fact, Irisin treatment protected hippocampal neurons against injury and cell death in cerebral ischemia models through different signaling pathways and mechanisms such as Akt, ERK1/2, Notch, TLR4/MyD88 and by protecting BBB from disruption (Li et al., 2017; Guo et al., 2019; Jin et al., 2019; Yu Q. et al., 2020). FNDC5 expression in skeletal muscle and circulating Irisin levels were also decreased by cerebral ischemia (Yu Q. et al., 2020). In addition, different types of exercise have been shown to protect brain against ischemic insult. For example, acute treadmill running prior to stroke protected the brain by enhancing angiogenesis in rats (Pianta et al., 2019). Similarly, treadmill running for 14 days protected prefrontal cortex neurons against cerebral ischemic induced apoptosis (Pianta et al., 2019). Importantly, neutralizing Irisin in peripheral blood using an antibody, eliminated the positive effect that exercise had on neuroprotection against cerebral ischemia (Li et al., 2017).

### FNDC5/Irisin in Neuropsychiatric Disorders

Neuropsychiatric disorders such as depression, anxiety, and schizophrenia, are high prevalent (Kessler et al., 2005). Thus, finding successful treatments against them is one of the main current challenges in neuroscience research. Exercise has been introduced as a potential treatment for preventing and alleviating neuropsychiatric disorders (Swenson et al., 2020). As revealed by several recent studies, FNDC5/Irisin could act as mediator for the skeletal muscle-brain axis that could be used as a therapeutic avenue in neuropsychiatric disorders.

I.c.v. injection of Irisin decreases immobility time in tail suspension and forced swimming tests in male mice, change that could be interpreted as anti-depressant in animal models (Siteneski et al., 2018). Similarly, Irisin level in prefrontal cortex is decreased by chronic stress in male Sprague–Dawley rats. Specifically, Irisin levels were correlated with chronic stress-induced depression behavior and recombinant Irisin injection for 14 days suppresses depressive-like behavior through controlling glucose metabolism in prefrontal cortex (Wang and Pan, 2016). Further, a human study showed that post-stroke depression is associated with decreased serum Irisin levels (Tu et al., 2018). The expression of PGC-1α is decreased in the hippocampus of mice with depressive-like behavior (Fu et al., 2020). BDNF level in the hippocampus and peripheral blood is correlated negatively with the severity of depression in rodents and humans (Lee et al., 2007; Serra et al., 2018). As discussed in previous sections, exercise induced FNDC5/Irisin increases BDNF expression in different regions of the brain, especially hippocampus.

Irisin injection into the brain decreases anxiety-like behavior in male mice (Siteneski et al., 2018). Our own data has shown that acute stress-induced anxiety like behavior is suppressed by injection of Irisin into the hippocampus in male mice only, without affecting female mice (Jodeiri Farshbaf et al., 2020). Further studies have shown that Irisin level is reduced in serum in subjects diagnosed with anxiety disorders (Szilasi et al., 2017). This is relevant considering different studies showing that exercise can reduce anxiety (reviewed in Anderson and Shivakumar, 2013). Furthermore, PGC-1α and BDNF levels have also been altered in rodents with anxiety-like behavior (Govindarajan et al., 2006; Szalardy et al., 2018), and both PGC-1α and BDNF can have anxiolytic properties (Agrawal et al., 2014; Xie et al., 2019). Exercise also reduces panic disorder, which is a type of anxiety disorder, through increasing BDNF concentration in serum (Strohle et al., 2010).

## Discussion

Altogether, there is solid evidence of the exercise-induced functional connection between skeletal muscles and the brain, and FNDC5/Irisin is a likely mediator. The multiple endocrine actions of Irisin in the CNS highlight the importance of this circulating myokine in neuroprotection against different injuries and insults, including neurodegenerative disorders.

**FIGURE 4:**
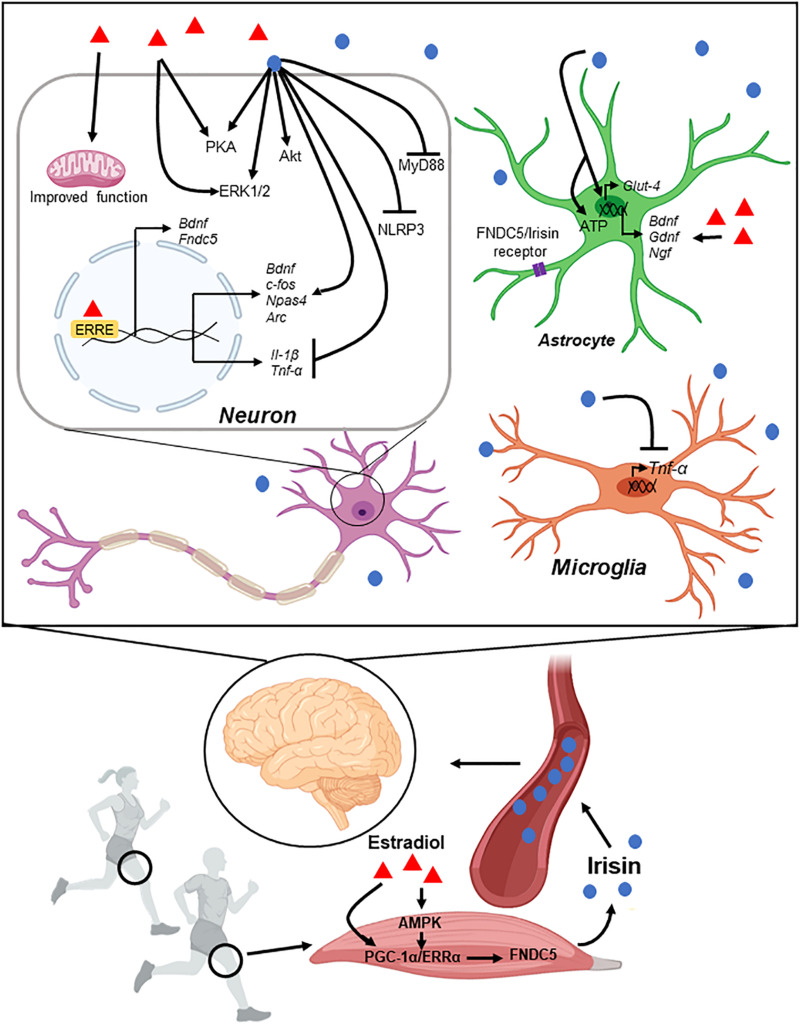
Schematic representation of muscle-brain connection through Irisin. Enhancement in FNDC5 expression by exercise leads to Irisin secretion into the peripheral blood. Irisin crosses the blood brain barrier and reaches different regions of the brain. Irisin controls gene expression and different signaling pathways in neurons and glial cells (see main text for details).

Different myokines are released from skeletal muscles in conditions of physical activity and several can reach the CNS, likely mediating the multiple beneficial effects associated with exercise (see Table 1). Irisin is a recently discovered myokine that is secreted form skeletal muscle during exercise and can cross the blood brain barrier. Irisin and its precursor, FNDC5, have important roles in development and regeneration of muscle, and other metabolic processes (Figure 4). However, only recently FNDC5/Irisin has been investigated as possible mediator of exercise-induced benefits for brain function. Specifically, Irisin release induces BDNF expression in the hippocampus leading to improvement in learning and memory, and protection against injuries such as ischemia, acute stress, and neurodegenerative disorders such as AD. Intriguingly, several sex-depended differences have been described in the context of exercise effects on health and cognitive function, mirroring the specific effects of FNDC5/Irisin. These findings warrant the need for expanding our research to include sex as a biological variable in the context of exercise promoted neuroprotection.

One of the areas of potential therapeutic relevance is the discovery of the receptor for Irisin in the brain, either in neurons and/or glial cells, and the specific mechanisms underlying its neuroprotective effects. To date, the only FNDC5/Irisin receptor that has been characterized occurs in bone and adipose tissue cells (Kim et al., 2018). It is possible to speculate about a similar type of receptor present in the CNS and with that knowledge many possible pharmacological interventions can be devised. This could be greatly relevant for aging related conditions such as neurodegenerative diseases, including AD. In this context, the possible identification of Irisin as an important mediator of the “muscle-brain axis” could have several implications for therapeutics development. The results summarized in this review indicate that FNDC5/Irisin could potentially be used as mimetic of exercise, in cases for example that physical activity is not recommended or not possible. Moreover, understanding what roles FNDC5/Irisin play as exercise intermediary could further cement exercise as non-pharmacological alternative intervention for individuals at risk, and even general aging population. Furthermore, the current knowledge in the field strongly indicates that Irisin (and possibly other myokines) could be considered and explored as a biomarker of unhealthy aging and neurodegeneration. This could have large consequences for public health initiatives considering that AD and related dementias affect to a large group of the aging population, and while enormous efforts have been dedicated to find a cure, there is currently no available treatment.

## Author Contributions

MJF and KA devised the original plan for the review. MJF wrote the initial draft, sketched initial figures, and revised the final draft. KA edited first draft and figures and finalized the manuscript and figures. Both authors contributed to the article and approved the submitted version.

## Conflict of Interest

The authors declare that the research was conducted in the absence of any commercial or financial relationships that could be construed as a potential conflict of interest.

## Footnotes

1 https://fantom.gsc.riken.jp/data
